# Kinesin Family Member C1 (KIFC1/HSET) Underlies Aggressive Disease in Androgen Receptor-Low and Basal-Like Triple-Negative Breast Cancers

**DOI:** 10.3390/ijms242216072

**Published:** 2023-11-08

**Authors:** Nikita Jinna, Yate-Ching Yuan, Padmashree Rida

**Affiliations:** 1 Department of Population Science, City of Hope Comprehensive Cancer Center, Duarte, CA 91010, USA; 2 Department of Integrative Genomics and Bioinformatics, City of Hope Comprehensive Cancer Center, Duarte, CA 91010, USA; yyuan@coh.org; 3 Department of Science, Rowland Hall, Salt Lake City, UT 84102, USA; padmashreerida@rowlandhall.org

**Keywords:** androgen receptor, Hset, kinesin family member C1, quadruple-negative breast cancer, triple-negative breast cancer

## Abstract

Quadruple-negative breast cancer (QNBC) lacks traditional actionable targets, including androgen receptor (AR). QNBC disproportionately afflicts and impacts patients of African genetic ancestry. Kinesin family member C1 (KIFC1/HSET), a centrosome clustering protein that prevents cancer cells from undergoing centrosome-amplification-induced apoptosis, has been reported to be upregulated in TNBCs and African-American (AA) TNBCs. Herein, we analyzed KIFC1 RNA levels and their associations with clinical features and outcomes among AR-low and AR-high TNBC tumors in three distinct publicly available gene expression datasets and in the breast cancer gene expression database (bc-GenExMiner). KIFC1 levels were significantly higher in AR-low and basal-like TNBCs than in AR-high and non-basal-like TNBCs, irrespective of the stage, grade, tumor size, and lymph node status. KIFC1 levels were also upregulated in AR-low tumors relative to AR-high tumors among Black and premenopausal women with TNBC. High KIFC1 levels conferred significantly shorter overall survival, disease-free survival, and distant metastasis-free survival among AR-low and basal-like TNBC patients in Kaplan–Meier analyses. In conclusion, KIFC1 levels may be upregulated in AR-low tumors and, specifically, in those of African descent, wherein it may promote poor outcomes. KIFC1 may be an actionable cancer-cell-specific target for the AR-low TNBC subpopulation and could aid in alleviating racial disparities in TNBC outcomes.

## 1. Introduction

Breast cancer is the most common form of cancer that is diagnosed among women in the United States (US), excluding nonmelanoma skin cancer [1]. It ranks second in the US for cancer-related deaths among women overall; however, it is the leading cause of cancer-related deaths among Black/African-American (AA) women [2]. Additionally, under the age of 40, Black/AA women display higher incidence rates but, overall, a 40% higher mortality rate compared to non-Hispanic White/European-American (EA) women [1]. Furthermore, breast cancer negatively influences the quality of life of patients with pathologies such as lymphedema. This post-oncological pathological condition of the lymphatic vessels is studied with magnetic resonance imaging and is a chronic condition that greatly aggravates the state of health of patients [3,4,5,6].

The poor outcomes among AA women diagnosed with breast cancer can partly be attributed to the higher incidence of aggressive breast cancer subtypes among AA women. Breast cancers can be categorized into four major intrinsic molecular subtypes: luminal A (ER^+^/PR+/HER^−^), luminal B (ER^+^/PR^+^/^−^/HER2^+^ or ER^+^/PR^+^/HER^−^ with Ki67%≥15%), HER2-enriched (ER^−^/PR^−^/HER2^+^), and triple-negative breast cancer (ER^−^/PR^−^/HER2^−^; TNBC) [7]. These unique subtypes aid clinicians in risk stratification and clinical decision making for managing the disease.

TNBC is a significant underlying factor in the high rate of breast cancer mortality observed among AA women in the US. As a result of lacking the traditional breast cancer targets—ER, PR, and HER2—women with TNBC do not benefit from endocrine and HER2-targeted systemic therapies. Thus, chemotherapy is the conventional but cytotoxic form of treatment that is often administered to all women with TNBC, with no consideration being given to race or the underlying molecular driver of the tumor [8]. Furthermore, TNBC is innately the most aggressive form of breast cancer, as evidenced by its significantly higher rate of distant metastases and recurrences, shorter survival time, and the 40% mortality rate within the first 5 years of diagnosis [9,10]. On average, TNBC patients relapse between 19 and 40 months, compared to 35–67 months for non-TNBC patients [10]. Moreover, TNBC is the most heterogenous subtype, with up to six distinct molecular subtypes that have been identified, namely, the basal-like 1 and 2 (BL1 and BL2), immunomodulatory (IM), mesenchymal (M), mesenchymal stem-like (MSL), and luminal androgen receptor (LAR) subtypes, according to one classification system [11]. These TNBC subtypes differ in aggressiveness, with the basal-like (BL1 and BL2) and IM subtypes conferring a poorer prognosis than that of the LAR subtype, which displays a more favorable outcome [11]. TNBC occurs more frequently in younger women (under the age of 40) and occurs at a rate that is two times higher in Black/AA women than in White/EA women, resulting in a stark racial disparity in breast cancer outcomes [1,12]. Furthermore, Black/AA women tend to present with more aggressive TNBC molecular subtypes, such as basal-like and IM [13,14].

QNBC has emerged as a newly recognized subtype of TNBC. In addition to lacking ER, PR, and HER2 expression, QNBCs also lack androgen receptor (AR) expression, which can be targeted pharmacologically. Approximately, 65–88% of TNBC lacks AR expression, suggesting that the vast majority of TNBC patients are not susceptible to AR-targeted therapies [15]. Furthermore, several studies have reported that low AR expression in TNBC confers a more aggressive disease course than that of AR-positive TNBC, including a younger age at diagnosis and shorter progression-free and overall survival [16,17,18,19,20,21,22]. QNBC also exhibits predominantly basal-like features and, thus, has been linked to the highly aggressive basal-like molecular phenotype [14,23]. Consistently with the other aggressive subtypes, QNBC disproportionately afflicts and impacts women of African descent, thus contributing to the observed racial disparity in breast cancer outcomes. Multiple groups have reported lower AR RNA and protein expression in Black/AA women than in White/EA women with TNBC [14,23,24]. Furthermore, Black/AA women with basal-like AR-negative TNBC experience significantly shorter progression-free survival than White/EA women do [14]. Hence, there is an urgent need for actionable targets for QNBC and, specifically, QNBCs that occur in women of African ancestry.

Kinesin family member C1 (KIFC1) is a minus-end directed microtubule binding protein that promotes the survival of cancer cells burdened with extra centrosomes or centrosome amplification [25,26]. The centrosome organizes the microtubules for proper bipolar cell division; thus, when centrosome number fidelity is compromised (i.e., when there are more than two centrosomes per cell), cell death can ensue because of mitotic catastrophe or multipolar mitotic cell divisions. KIFC1 can interfere with the activation of this cellular death program via crosslinking and by sliding microtubules to cluster extra centrosomes at opposite poles of the cell to allow cancer cells to undergo a “pseudo-bipolar” spindle mitosis. Centrosome clustering by KIFC1 also facilitates single-chromosome mis-segregation during these pseudo-bipolar mitoses and leads to a “tolerable and persistent” level of aneuploidy, which engenders karyotype reshuffling and clonal evolution of tumor cell populations to increase intratumoral heterogeneity. Thus, KIFC1 facilitates the production and survival of genomically unstable cancer cells, which can increase the pool of aggressive cellular clones. KIFC1 is essential for the viability of cancer cells with supernumerary centrosomes but not for healthy cells with a conserved number of centrosomes, suggesting that targeting KIFC1 proffers a highly selective and “cytofriendly” therapeutic strategy [27,28].

In recent years, KIFC1 has surfaced as an aggressiveness biomarker for breast cancer [29,30]. It is overexpressed in primary breast cancer compared to matched normal breast tissue [28]. Nuclear KIFC1 levels have been linked to advanced tumor grade and poorer overall- and progression-free survival in breast cancer [28]. In TNBC, nuclear KIFC1 expression levels are elevated compared to those in non-TNBC, and this imparts aggressive phenotypes to TNBC, such as enhanced cell cycle kinetics, resistance to apoptosis by stabilizing survivin, enhanced centrosome clustering ability, and taxane resistance [28,31]. Additionally, KIFC1 is in the top 1% of genes that are (a) upregulated in TNBC in relation to non-TNBC, (b) associated with p53 mutation, and (c) associated with a survival rate of <5 years [30,32]. Nuclear KIFC1 expression levels have been shown to be an independent prognostic biomarker of aggressive TNBC in Black/AA but not White/EA TNBC patients [27]. Since KIFC1 is associated with aggressive TNBC and Black/AA TNBC biology, dysregulated expression of KIFC1 may be both a poor prognostic biomarker and a highly selective, efficacious, and actionable target for QNBC disease.

In this multi-cohort study, we evaluated differential KIFC1 gene expression levels among AR-low and AR-high TNBCs and the associations of KIFC1 levels with demographic and clinical disease characteristics. We provide early evidence suggesting that KIFC1 may be associated with the aggressive tumor biology of AR-low TNBCs. Our preliminary evidence also suggests that KIFC1 could improve disease risk stratification for AR-low and basal-like TNBC patients and could serve as a promising target for therapeutic intervention in QNBC. Our work is unique in that it validates a potential QNBC biomarker in three separate and independent databases. Additionally, our initial findings support that KIFC1 targeting could aid in improving TNBC outcomes among Black/AA women and reduce the overall racially disparate burden in breast cancer.

## 2. Results

### 2.1. AR-Low Tumors Display Aggressive Clinical Features Compared to AR-High Tumors in TNBC

Low expression levels of AR in TNBC patients have been linked to unfavorable clinical characteristics, as well as younger age and AA ethnicity. We sought to verify these prior findings across all three publicly available datasets (TCGA, METABRIC, and RODY). As anticipated, AR-low tumors exhibited poorer clinical features than those of AR-high tumors, as displayed in Table 1, Table 2 and Table 3. The AR-low subgroup exhibited a greater proportion of basal-like tumors than the AR-high subgroup in both TCGA (94.74% vs. 51.72%) and METABRIC (63.33% vs. 37.58). AR-low tumors also displayed a higher clinical grade than that of AR-high tumors in the METABRIC (91.22% vs. 82.43%) and RODY (94% vs. 48%) datasets. Regarding patient demographics, the median age at diagnosis was found to be significantly lower among AR-low tumors than among AR-high tumors in METABRIC (54 vs. 57). 

### 2.2. KIFC1 RNA Levels Are Upregulated in AR-Low vs. AR-High TNBC Tumors

**Figure 1:**
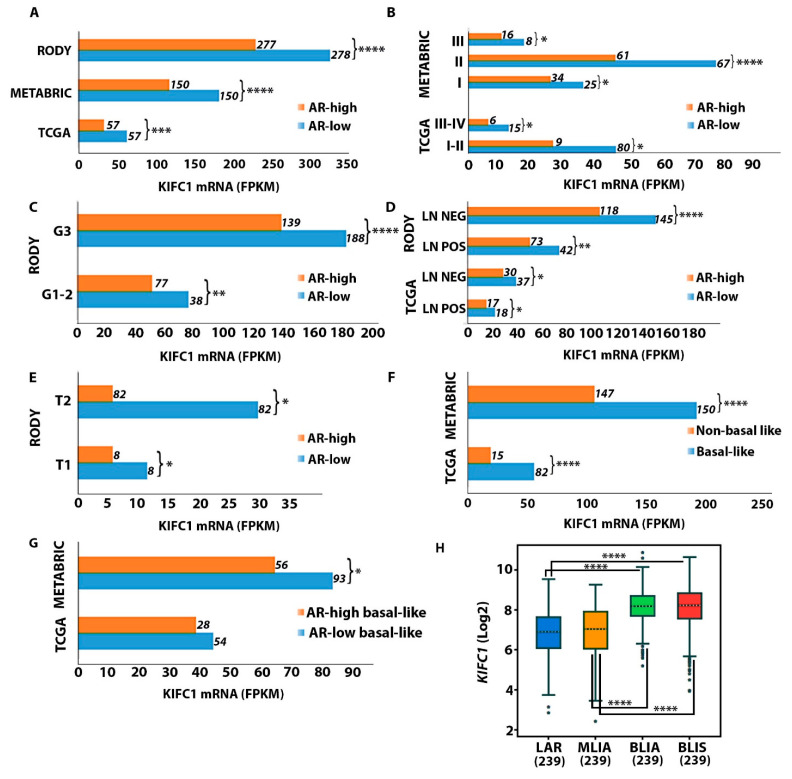
Comparison of KIFC1 RNA levels between the AR-low and AR-high TNBC samples across multiple independent databases. Differences in KIFC1 RNA levels between AR-low and AR-high TNBC tumors overall (**A**), stage-wise (**B**), grade-wise (**C**), according to lymph node status (**D**), and according to tumor size (**E**) in the TCGA, METABRIC, and RODY gene expression datasets. Differences in KIFC1 RNA levels between basal-like and non-basal-like TNBC tumors in the TCGA and METABRIC datasets (**F**). Differences in KIFC1 RNA levels between AR-low and AR-high basal-like tumors in the TCGA and METABRIC datasets (**G**). Differences in KIFC1 RNA levels among three different TNBC subtypes in the bc-GenExMiner dataset (**H**). *p* < 0.05 is considered significant. Numbers alongside the bars represent the number of cases. *, <0.05; **, <0.01; ***, <0.001; ****, <0.0001. Abbreviations: LAR, luminal androgen receptor; MLIA, mesenchymal-like immune-activated; BLIA, basal-like immune-activated; BLIS, basal-like immune-suppressed.

To determine if KIFC1 is upregulated in QNBC, we compared KIFC1 RNA expression levels between the AR-low and AR-high TNBC samples across three different publicly available datasets, namely, RODY, METABRIC, and TCGA (Figure 1). We discovered that the KIFC1 RNA levels were significantly higher in the AR-low samples than in the AR-high samples in the RODY, METABRIC, and TCGA datasets (Figure 1A). KIFC1 levels were also upregulated in the AR-low samples compared to the AR-high samples in each of the stage, grade, lymph node status, and tumor size categories (Figure 1B–E). Since basal-like TNBC has similar features to those of the QNBC phenotype, we also compared the KIFC1 RNA levels between available basal-like and non-basal-like TNBC samples in the TCGA and METABRIC datasets. The KIFC1 RNA levels were significantly higher in the basal-like samples than in the non-basal-like TNBC samples in both the TCGA and METABRIC datasets (Figure 1F). In addition, the KIFC1 RNA levels were found to be higher in the AR-low subgroups than in the AR-high subgroups among basal-like TNBC patients, suggesting that, irrespective of basal-like status, KIFC1 is associated with low AR expression levels in TNBC (Figure 1G). We lastly compared the KIFC1 RNA levels among four different TNBC molecular subtypes identified in bc-GenExMiner, namely, the luminal androgen receptor (LAR), mesenchymal-like immune activated (MLIA), basal-like immune suppressive (BLIS), and basal-like immune activated (BLIA) subtypes. Our analysis showed that the KIFC1 RNA levels were notably higher in both basal-like TNBC subtypes, which were low in AR RNA levels, compared to the LAR subtype, which was enriched in AR expression, as well as compared to the MLIA subtype (Figure 1E). Table 1, Table 2 and Table 3 also show that the median expression levels of KIFC1 were significantly higher among AR-low than among AR-high tumors across all databases.

Multiple groups have revealed that AR RNA expression is directly correlated with AR protein expression [33,34]. Thus, we analyzed if KIFC1 RNA levels were correlated with AR RNA levels, which could reflect KIFC1 expression levels based on levels of the functional protein product of AR (Figure S1). Consistently with the results above, KIFC1 RNA expression was negatively correlated with AR RNA levels among the TNBC samples in TCGA (Figure S1A), METABRIC (Figure S1B), RODY (Figure S1C), and bc-GenExMiner datasets (Figure S1D). Furthermore, among basal-like TNBC patients, KIFC1 RNA expression was negatively correlated with AR RNA levels in the METABRIC (Figure S1E) and bc-GenExMiner datasets (Figure S1F). Collectively, this evidence further suggests that QNBCs may have upregulated expression of KIFC1 relative to AR-positive TNBCs.

### 2.3. KIFC1 RNA Is Upregulated in Black/AA and Premenopausal AR-Low Tumors

**Figure 2:**
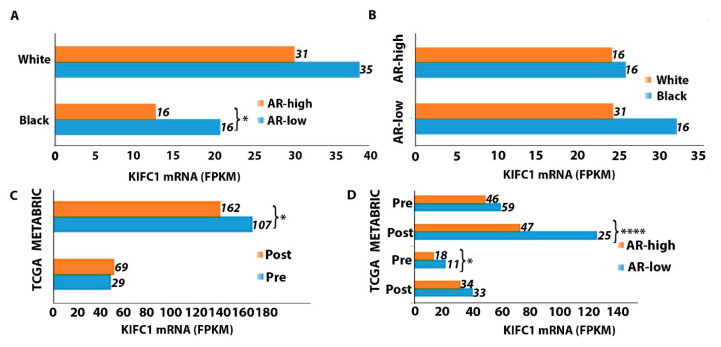
Comparison of KIFC1 RNA levels between AR-low and AR-high TNBC tumors according to racial and menopausal status. Differences in KIFC1 RNA expression between AR-low and AR-high TNBC tumors according to racial status (**A**). Differences in KIFC1 RNA expression between Black and White patients with AR-low and AR-high TNBC tumors (**B**). Differences in KIFC1 RNA expression between pre- and postmenopausal TNBC tumors in the TCGA and METABRIC datasets (**C**). Differences in KIFC1 RNA expression between AR-low and AR-high TNBC tumors among pre- and postmenopausal patients in the TCGA and METABRIC datasets (**D**). *p* < 0.05 is considered significant. *, <0.05; ****, <0.0001.

Since younger and Black/AA women are disproportionately impacted by QNBC, we analyzed differences in KIFC1 RNA levels between AR-low and AR-high TNBC samples according to racial and menopausal status (Figure 2). Among Black/AA patients, the AR-low subgroup exhibited significantly higher KIFC1 expression relative to the AR-high subgroup (Figure 2A). This difference was not pronounced in the White/EA population, however (Figure 2A). In both the AR-low and AR-high subgroups, Black/AA women displayed higher KIFC1 expression than that of White/EA women, but this difference was not statistically significant (Figure 2B). Regarding menopausal status, premenopausal samples displayed significantly higher KIFC1 expression than that of the postmenopausal samples in the METABRIC database (Figure 2C). Furthermore, among the premenopausal samples, the AR-low subgroup exhibited significantly higher KIFC1 expression than that in the AR-high subgroup in the TCGA database (Figure 2D). However, in the METABRIC database, this difference was observed among postmenopausal samples (Figure 2D).

### 2.4. High KIFC1 Levels Are Associated with a Poorer Prognosis among AR-Low Patients

**Figure 3:**
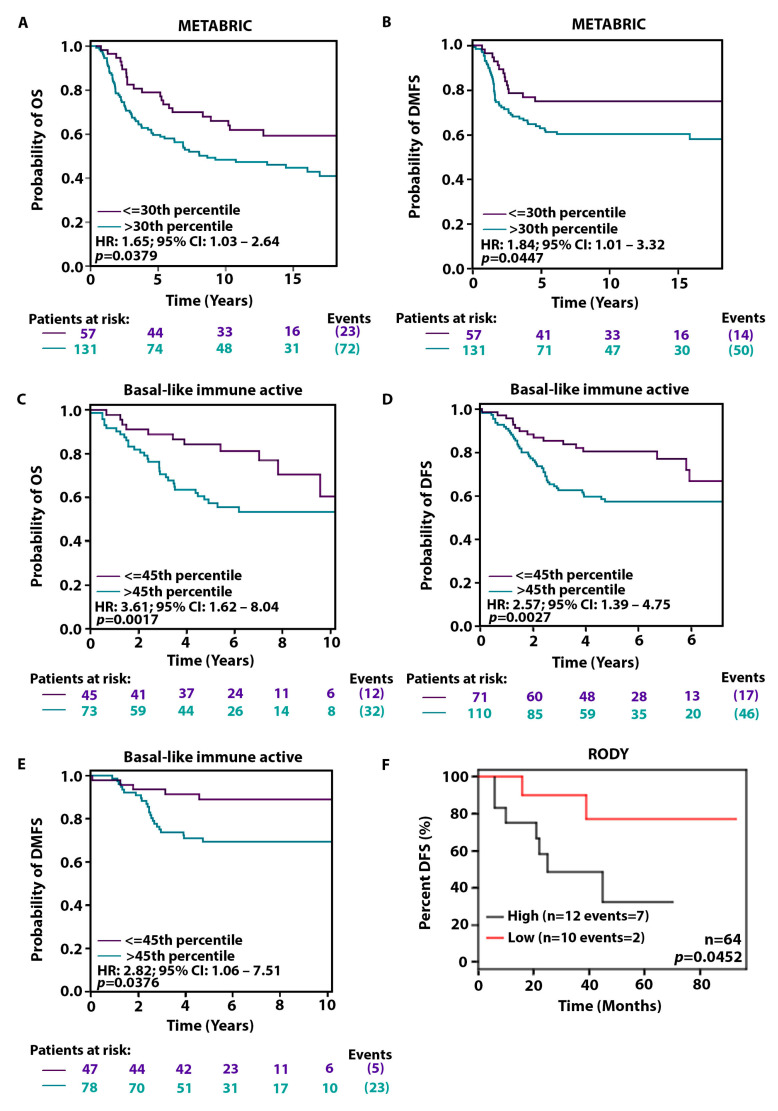
Association between KIFC1 expression levels and clinical outcomes in women with AR-low and basal-like TNBC. Kaplan–Meier associations of KIFC1 RNA levels with OS (**A**) and DMFS (**B**) among basal-like TNBC patients in the METABRIC bc-GenExMiner dataset. Kaplan–Meier associations of KIFC1 RNA levels with OS (**C**), DFS (**D**), and DMFS (**E**) among basal-like immune-active TNBC patients in GenExMiner. Kaplan–Meier association of KIFC1 RNA levels with DFS among AR-low basal-like TNBC patients in the RODY dataset (**F**). Optimal cut-offs were referred to for all Kaplan–Meier analyses.

Since we have shown that KIFC1 RNA levels are notably higher in AR-low TNBCs than in AR-high TNBCs, next, we investigated whether KIFC1 could be driving the poorer prognosis observed in QNBCs relative to AR-positive TNBCs. Hence, we analyzed the influence of KIFC1 on survival outcomes. In Kaplan–Meier analyses, we discovered that high KIFC1 expression levels were significantly associated with poorer clinical outcomes (Figure 3). High KIFC1 levels conferred notably shorter overall survival (OS) (Figure 3A) and distant metastasis-free survival (DMFS) (Figure 3B) among basal-like TNBC patients and significantly shorter OS (Figure 3C), disease-free survival (DFS) (Figure 3D), and DMFS (Figure 3E) among basal-like immune-active TNBC patients. In the RODY dataset, high KIFC1 expression also conferred significantly shorter DFS among AR-low basal-like TNBC patients (Figure 3F). Collectively, these findings indicate that high KIFC1 levels may drive an aggressive course of disease among QNBC patients.

### 2.5. Genes Driving Centrosome Amplification Are Upregulated in AR-Low TNBCs Compared to AR-High TNBCs

As previously mentioned, KIFC1 prevents cancer cells burdened with extra centrosomes from undergoing mitotic-error-induced cell death by clustering these extra centrosomes at opposite poles of the cell during mitosis. Therefore, KIFC1 is often upregulated alongside centrosome amplification [35]. Thus, we correlated the expression of genes whose dysregulation is linked to the onset of centrosome amplification with AR mRNA expression among TNBCs in bc-GenExMiner [36]. We discovered highly significant negative correlations of 17 key centrosome-amplification-driving genes with AR gene expression (Table S1). Thus, centrosome amplification and KIFC1 are likely to be co-upregulated among AR-low TNBC tumors. We then evaluated differences in the mean expression of the top 12 genes involved in centrosomal dysregulation that were negatively correlated with AR between the four different TNBC subtypes (Figure S2). Similarly to the findings with KIFC1, the mean expression of each centrosome-amplification-driving gene was significantly higher in each of the basal-like subtypes (BLIA and BLIS) relative to the LAR subtype and in comparison with the MLIA subtype. This finding further suggests that centrosome amplification is more rampant among AR-low TNBC tumors than among AR-high TNBC tumors. Lastly, we exploited the online KM Plotter tool to determine if centrosome-amplification-driving genes also promoted poorer outcomes among AR-low TNBC patients. We discovered that the mean expression of all 17 centrosome-amplification-driving genes, with the exception of CDK1, conferred significantly shorter recurrence-free survival among BL1 TNBC tumors, but not among LAR TNBC tumors (Figure S3).

## 3. Discussion

The management and treatment of aggressive breast cancers, such as TNBCs, continue to challenge clinicians. These “targetless” subtypes offer patients a dismal number of targeted treatment options. Since QNBCs also lack the potential TNBC target, AR, there is an urgent need to identify actionable biomarkers in this unique subgroup of breast cancers that disproportionately impacts individuals of African ancestry. In this study, we illuminated the potential role of an identified TNBC and Black/AA TNBC biomarker, KIFC1, as a biomarker of aggressive AR-low disease.

Consistently with prior evidence, the AR-low group displayed unfavorable disease characteristics across three independent databases compared to the AR-high group. Specifically, AR-low tumors presented with more basal-like features and more advanced grade compared to AR-high tumors. Previously, the lack of AR expression in TNBC was associated with the expression of basal markers (i.e., CK15, CK14, CK5/6, nestin), proliferation markers (Ki-67), a basal-like phenotype (CK5^+^ and/or nestin^+^), and basal-like signatures (BL1 and BL2), which were linked to a poor prognosis, including short overall and breast-cancer-specific survival [14,37,38,39]. Overwhelming evidence, including that in the Carolina Breast Cancer Study (CBCS), has shown a strong link between low AR levels and advanced tumor grade in TNBC [19,24,37,40,41]. Moreover, consistently with previous reports, such as that from the CBCS, the AR-low tumors in our study presented at a younger age than the AR-high tumors did [14,24,38,42]. Collectively, this evidence supports the urgent need to identify actionable biomarkers for QNBC.

KIFC1 is a cancer-cell-specific protein that aids in the persistent survival and proliferation of tumor cells by averting spindle-multipolarity-induced cellular death. Elevated KIFC1 levels have been implicated in the onset and progression of several cancer types, including aggressive breast cancer and TNBC. Hence, we investigated a potential role of KIFC1 in QNBC by analyzing differences in KIFC1 RNA levels among TNBC tumors according to AR status. In all three publicly available databases, we discovered that KIFC1 expression was notably upregulated among AR-low and basal-like tumors relative to AR-high and non-basal-like tumors, respectively, among TNBC samples, irrespective of the grade, stage, lymph node status, and tumor size. We also discovered in bc-GenExMiner that KIFC1 levels were significantly elevated in the BLIS and BLIA TNBC subtypes relative to the AR-enriched LAR TNBC subtype. Exclusively among basal-like tumors, KIFC1 levels remained elevated among AR-low samples relative to those among AR-high samples. Furthermore, KIFC1 levels were negatively correlated with AR levels among TNBC in all of the examined datasets. These findings corroborate those of prior reports revealing upregulated levels of proliferation and DNA damage signaling markers in the AR-negative and basal-like subgroup of TNBC tumors. For example, QNBC tumors have been reported to be upregulated in Ki-67, EGFR, VEGF, cKIT, TOPO2A, E2F1, and CDK6, and downregulated in PTEN and HER4 [23]. Thus, KIFC1 may represent an actionable, cancer-cell-specific biomarker that is reflective of proliferative QNBC tumor biology.

Consistently with prior reports that centrosome amplification triggers the upregulation of KIFC1 to circumvent cell death, we also observed that genes whose dysregulation can promote centrosome amplification were negatively correlated with AR expression and were significantly upregulated in the basal-like TNBC subtypes relative to the LAR TNBC subtype. Furthermore, these centrosome-amplification-driving genes were found to promote shorter recurrence-free survival among basal-like TNBC patients but not among AR-enriched TNBC patients. These findings suggest that KIFC1 is likely to be upregulated among AR-low/basal-like TNBC tumors as a result of the increased centrosomal aberrations present among this subgroup relative to AR-high/non-basal-like TNBC tumors. Thus, cancer-cell-specific agents targeting both KIFC1 and/or centrosome amplification in AR-low or basal-like TNBCs may effectively aid in the eradication of this tumor type.

Mounting evidence shows that young and Black/AA women predominantly develop AR-negative TNBC. We similarly observed a significantly greater prevalence of AR-low tumors among premenopausal women with TNBC. In addition, among premenopausal tumors, KIFC1 levels were notably higher in AR-low TNBC than in AR-high TNBC, thus providing evidence that KIFC1 may be a potential actionable target in the treatment of young women with AR-low TNBC. Furthermore, in Black/AA women with TNBC only, the KIFC1 levels were significantly higher among AR-low tumors than among AR-high tumors. This finding suggests that KIFC1 may be a biomarker that is specific for Black/AA QNBC and could be a promising therapeutic target for this patient subpopulation, which is disproportionately afflicted and impacted by QNBC.

Overexpression of KIFC1 has been linked to advanced tumor grade, taxane resistance, and poor survival rates in breast cancer [28,29,43,44]. However, in TNBC, KIFC1 has been shown to specifically confer a poor prognosis in Black/AA patients; high nuclear KIFC1 levels have been associated with significantly shorter OS, progression-free survival (PFS), and DMFS in Black/AA women—but not White/EA women—with TNBC in multivariable Cox models [27]. Furthermore, KIFC1 knockdown and inhibition impaired migration and cell viability, respectively, in Black/AA TNBC cells but not White/EA TNBC cells [27,45]. These unique findings have rendered KIFC1 as a robust biomarker of Black/AA TNBC and as a promising actionable target for this TNBC subpopulation. In this study, we confirmed via Kaplan–Meier analyses that high KIFC1 levels are associated with shorter OS, DFS, and DMFS among basal-like and basal-like immune-active TNBC patients in multiple independent databases. In the RODY database, high KIFC1 levels conferred shorter DFS among AR-low basal-like TNBC patients. Since the tumor biology in both QNBC and AR-low TNBC is characterized by basal-like features and an active immune profile, this discovery (a) furthers our understanding of the molecular drivers of QNBC’s aggressive clinical behavior and (b) advances upregulated KIFC1 as a potential therapeutic target for AR-low and Black/AA TNBC. The outcomes for patients in these high-need subgroups can only be improved if prognostic tools and treatment modalities are precisely tailored to the molecular drivers of aggressiveness specific to these groups. Our in silico findings may slightly differ from the aforementioned IHC-based study results due to the absence of nuclear KIFC1 information, which proffers valuable prognostic information.

The absence or lack of a fourth actionable target in breast cancer, AR, has further complicated the management and treatment of TNBC, for which chemotherapy is the mainstay of treatment. There is a paucity of biomarkers that can guide clinical decision making for this unique subgroup of TNBC and can circumvent these challenges. Our investigation uncovered an alternative actionable target that is highly upregulated in TNBC tumors that are not enriched for AR expression and confers poor survival in this patient subpopulation. This discovery suggests that TNBC, which are not driven by AR signaling, could be pharmacologically targeted in the clinic with a cancer-cell-specific treatment strategy in lieu of cytotoxic chemotherapy. Our study also highlighted the fact that TNBC is, indeed, notoriously heterogeneous; there are substantial biological differences between “AR-low” TNBC and “AR-high” TNBC, including the higher expression of KIFC1 in the “AR-low” subgroup. Our findings further illuminate that even the label “AR-low TNBC” flattens the landscape of these tumors and masks their heterogeneity because these tumors can be further stratified into KIFC1-high cases with a poorer prognosis and a high risk of progression and a KIFC1-low subgroup with a better prognosis. These data support the potential clinical benefit of targeting the druggable molecule KIFC1 as an adjuvant for chemotherapy in all high-risk, high-KIFC1, AR-low TNBCs. Further investigation of nuclear KIFC1 expression levels in racially distinct AR-negative TNBC tissue and their association with disease characteristics and clinical outcomes will be necessary for the establishment of KIFC1 as a robust actionable biomarker of QNBC. Additionally, investigation of the responses to KIFC1-targeted drugs, such as CW069 and AZ82, in racially distinct QNBC in vitro and in vivo will also be critical in pushing KIFC1-targeted therapy to the forefront of the management and treatment of QNBC and Black/AA QNBC.

## 4. Materials and Methods

### 4.1. Study Populations

We downloaded RNA-seq output data from 1095 primary breast tumor specimens from The Cancer Genome Atlas (TCGA) database, 2509 from the Molecular Taxonomy of Breast Cancer International Consortium (METABRIC) database, and 579 from the GSE31519 (RODY) database by using the QIAGEN OmicSoft Studio V11.7 software. The RNA-seq output from the RODY database was log2-transformed to normalize the gene expression values for each sample (https://www.ncbi.nlm.nih.gov/geo/query/acc.cgi?acc=gse31519, accessed on 11 November 2022). The accompanying clinical data, including survival outcomes and tumor characteristics, were downloaded for each sample. Only the RNA-seq data from the TNBC samples for each cohort were analyzed for this study. Only RNA-seq data from TNBC tumors with AR and KIFC1 were included in the analyses. The analyzed TNBC samples were categorized into AR-low and AR-high subgroups according to a 50% quantile threshold. To further support our analyses of publicly available databases, we exploited Affymetrix microarray TNBC primary tumor data from the online Breast Cancer Gene-Expression Miner v4.8 (bc-GenExMiner v4.8) and Kaplan–Meier (KM) Plotter tools.

### 4.2. Statistical Analyses

Wilcoxon rank sum tests were performed to analyze differences in KIFC1 RNA expression between two groups (i.e., AR-low vs. AR-high, basal-like vs. non-basal-like). The Dunnett–Tukey–Kramer test was performed to determine differences in KIFC1 gene expression between the different TNBC subtypes. Pearson correlation coefficients were computed to determine the correlation between AR and KIFC1 gene expression. Survival curves and Kaplan–Meier curves were constructed to analyze the univariate associations of KIFC1 gene expression with survival outcomes according to the optimal cut-point. Log-rank tests were performed to determine *p*-values for statistical differences in the survival distributions. For continuous variables, the median and range are presented. *p*-values were computed using the Wilcoxon rank sum test for continuous variables and Fisher’s exact test for categorical variables. Patient counts and percentages are presented for categorical variables. *p*-values are two-sided with a significance level of 0.05. The analysis was performed with SAS 9.4 (SAS Institute Inc., Cary, NC, USA) and R version 4.2.0 (R Foundation, Vienna, Austria; https://www.r-project.org, accessed on 5 December 2022).

In the bc-GenExMiner v4.8 program, Wilcoxon rank sum tests were performed to analyze differences in KIFC1 RNA expression between two groups (i.e., AR-low vs. AR-high, basal-like vs. non-basal-like). The Dunnett–Tukey–Kramer test was performed to determine differences in KIFC1 gene expression between the different TNBC subtypes. Pearson correlation coefficients were computed to determine the correlation between AR and KIFC1 gene expression. Survival curves and Kaplan–Meier curves were constructed to analyze univariate associations of KIFC1 gene expression with survival outcomes according to the optimal cut-point. Log-rank tests were performed to determine *p*-values for statistical differences in the survival distributions.

## Figures and Tables

**Table 1:** Characteristics of TNBC patients in TCGA according to AR levels.

	AR Higher 50% (N = 58) ^1^	AR Lower 50% (N = 57)	Total (N = 115)	*p*-Value ^2^
KIFC1	22.9 (1.04–86.62)	30.02 (10.1–72.1)	26.49 (1.04–86.62)	0.0029
AR	2.22 (0.62–56.7)	0.28 (0.03–0.58)	0.62 (0.03–56.71)	Not applicable
Age At Diagnosis (years)	55 (29–90)	53 (38–82)	54 (29–90)	0.871
Race				0.690
Asian	4 (7)	4 (7)	8 (7)	
Black or African American	17 (29)	15 (26)	32 (28)	
White	32 (55)	36 (63)	68 (59)	
NA	5 (9)	2 (4)	7 (6)	
TNM_T_stage				1.000
M0	49 (84)	49 (86)	98 (85)	
M1	1 (2)	1 (2)	2 (2)	
MX	8 (14)	7 (12)	15 (13)	
TNM_N_stage				0.764
N0	36 (62)	38 (67)	74 (64)	
N1	12 (21)	13 (23)	25 (22)	
N2	7 (12)	5 (9)	12 (10)	
N3	3 (5)	1 (2)	4 (3)	
Overall_stage				0.366
Missing	3 (0)	4 (0)	7 (6.1)	
Stage I	12 (22)	7 (13)	19 (17)	
Stage II	32 (58)	39 (74)	71 (62)	
Stage III	10 (18)	6 (11)	16 (14)	
Stage IV	1 (2)	1 (2)	2 (2)	
Tumor subtype				<0.001
Basal	30 (52)	54 (95)	84 (73)	
Other	28 (48)	3 (5)	31 (27)	
Lymph-Node-Positive Number Group				0.685
0	31 (63)	37 (67)	68 (59)	
≥1	18 (37)	18 (33)	36 (31)	
Missing	9 (0)	2 (0)	11 (10)	
Lymph-Node-Positive Number	0 (0–14)	0 (0–10)	0 (0–14)	0.509
Menopause Status				0.061
Perimenopausal	1 (2)	4 (7)	5 (4)	
Postmenopausal	36 (62)	33 (58)	69 (60)	
Premenopausal	19 (33)	11 (19)	30 (26)	
Indeterminate	0 (0)	2 (4)	2 (2)	
NA	2 (4)	7 (12)	9 (8)	

^1^ Median (Range); n (%), ^2^ Wilcoxon rank sum test for continuous variables; Fisher’s exact test for categorical variables.

**Table 2:** Characteristics of TNBC patients in METABRIC according to AR levels.

	AR Higher 50% (N = 149) ^1^	AR Lower 50% (N = 150)	Total (N = 299)	*p*-Value ^2^
KIFC1	7.30 (5.53–9.14)	7.94 (6–10)	7.69 (5.53–9.68)	<0.001
AR	6.62 (5.88–10.16)	5.67 (5.22–5.87)	5.87 (5.22–10.16)	Not applicable
Age at Diagnosis (years)	57 (27–96)	54 (28–85)	56 (27–96)	0.032
Menopausal time point				0.127
Post	102 (69)	90 (60)	192 (64)	
Pre	47 (32)	60 (40)	107 (36)	
PAM50 Subtype				<0.001
Basal	56 (38)	95 (63)	151 (51)	
Others	93 (62)	55 (37)	148 (50)	
The Nottingham Prognostic Index (NPI)	4 (2–6)	4 (1–6)	4 (1–6)	0.820
Grade				0.026
Grade 1 or 2	26 (18)	13 (9)	39 (13)	
Grade 3	122 (82)	135 (91)	257 (86)	
Missing	1 (0)	2 (0)	3 (1)	
Tumor size (mm)	22 (1–182)	25 (0–84)	25 (0–182)	0.552
Tumor Stage				0.098
1	35 (24)	27 (18)	62 (21)	
2	62 (42)	68 (45)	130 (43)	
3	17 (11)	8 (5)	25 (8)	
null	35 (23)	47 (31)	82 (27)	
Chemotherapy				0.453
No	74 (50)	68 (45)	142 (47)	
Yes	75 (50)	82 (55)	157 (53)	

^1^ Median (Range); n (%), ^2^ Wilcoxon rank sum test for continuous variables; Fisher’s exact test for categorical variables.

**Table 3:** Characteristics of TNBC patients in the RODY dataset according to AR levels.

Variables	AR Lower 50%, N = 278 ^1^	AR Higher 50%, N = 278 ^1^	Overall, N = 556 ^1^	*p*-Value ^2^
AR	−0.00285 (−0.01445, −0.00155)	0.00015 (−0.00155, 0.00837)	−0.00155 (−0.01445, 0.00837)	Not applicable
KIFC1	0.003 (−0.009, 0.009)	0.001 (−0.009, 0.007)	0.002 (−0.009, 0.009)	<0.001
KIFC1 Quartile				<0.001
KIFC1 Q1	36 (13)	103 (37)	139 (25)	
KIFC1 Q2	66 (24)	73 (26)	139 (25)	
KIFC1 Q3	82 (29)	57 (21)	139 (25)	
KIFC1 Q4	94 (34)	45 (16)	139 (25)	
Age at diagnosis (years)	50 (28, 82)	53 (29, 88)	51 (28, 88)	0.003
Missing	53	77	130	
Lymph node status				<0.001
Negative	146 (77)	118 (61)	264 (69)	
Positive	43 (23)	74 (39)	117 (31)	
Missing	89	86	175	
Tumor size				>0.999
Up to 1 cm	8 (25)	8 (25)	16 (25)	
>1 cm	24 (75)	24 (75)	48 (75)	
Missing	246	246	492	
Histological grade				<0.001
Grade 1 or 2	39 (17)	78 (36)	117 (26)	
Grade 3	189 (83)	139 (64)	328 (74)	
Missing	50	61	111	
Chemotherapy treatment				0.476
No	3 (9.1)	6 (18)	9 (13)	
Yes	30 (91)	28 (82)	58 (87)	
Missing	245	244	489	

^1^ Median (Range); n (%), ^2^ Wilcoxon rank sum test for continuous variables; Fisher’s exact test for categorical variables.

## Data Availability

Not applicable.

## Supplementary Materials

The following supporting information can be downloaded at: https://www.mdpi.com/article/10.3390/ijms242216072/s1.
