# Supplementary material for: Kinesin Family Member C1 (KIFC1/HSET) Underlies Aggressive Disease in Androgen Receptor-Low and Basal-Like Triple-Negative Breast Cancers
Source: Int J Mol Sci. 2023 Nov 8;24(22):16072. doi: 10.3390/ijms242216072 (PMC10671256; doi:10.3390/ijms242216072)
Supplement: Supplementary file 1 [file ijms-24-16072-s001.zip › ijms-2651717-supplementary.pdf]

Supplementary Materials

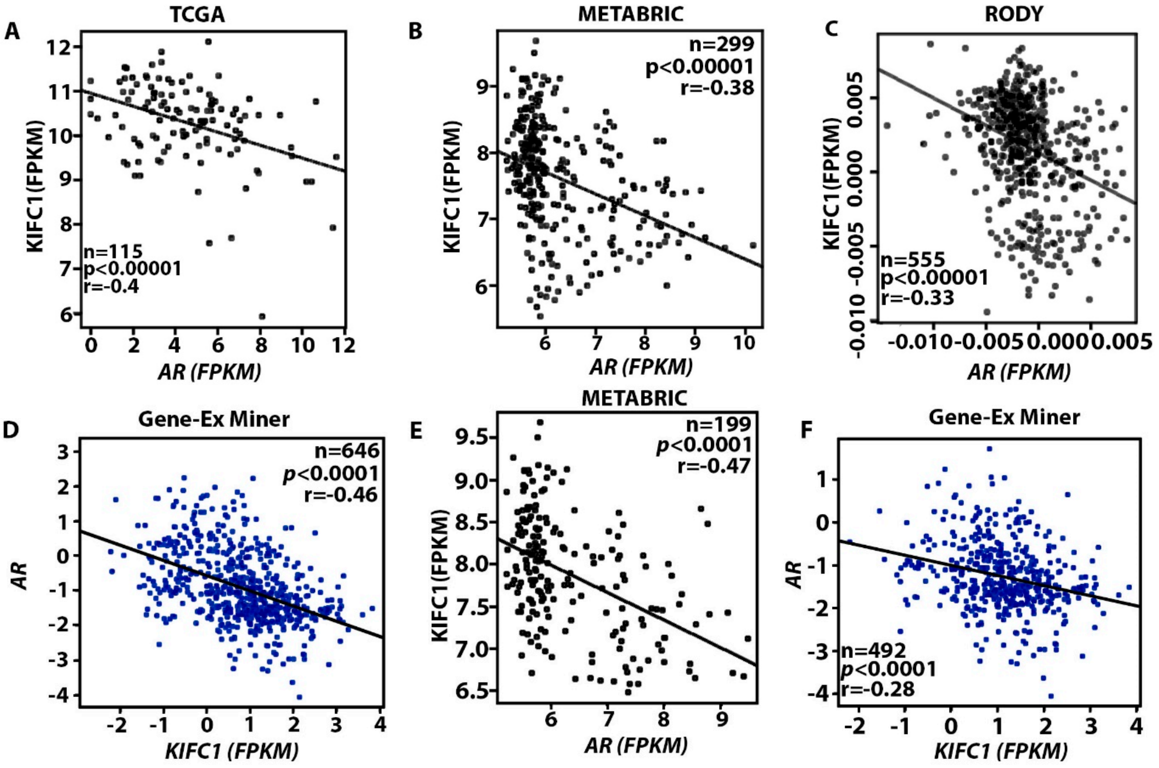

Figure S1. Correlation of KIFC1 RNA levels with AR RNA expression in TNBC and basal-like TNBC tumors cross multiple independent databases. Correlation of AR with KIFC1 RNA expression among TNBC tumors in the TCGA (A), METABRIC (B), RODY (C) and gene-x-miner (D) datasets. Correlation of AR with KIFC1 RNA expression among basal-like TNBC tumors in the METABRIC (E) and bc-GenExMiner (F). FPKM, fragments per kilobase exon model per million reads

Table S1. Correlation of centrosome-amplification driving genes with AR expression in TNBC tumors in bc-GenExMiner.

Gene	Pearson correlation coefficient (r)	p-value	Sample size (n)
PLK4	-0.41	<0.0001	937
PLK1	-0.39	<0.0001	937
CCNA2	-0.43	<0.0001	937
STIL	-0.39	<0.0001	937
NEK2	-0.41	<0.0001	937
E2F1	-0.12	0.0001	937
AURKA	-0.36	<0.0001	937
E2F2	-0.26	<0.0001	937
SASS6	-0.28	<0.0001	760

**CEP152**	**-0.25**	**<0.0001**	**937**
**CDK1**	**-0.34**	**<0.0001**	**937**
**LMO4**	**-0.35**	**<0.0001**	**980**
**CCNE2**	**-0.22**	**<0.0001**	**937**
**MYCN**	**-0.09**	**0.0030**	**980**
**TUBG1**	**-0.19**	**<0.0001**	**937**
**NDRG1**	**-0.22**	**<0.0001**	**980**
**PIN1**	**-0.01**	**0.6647**	**937**

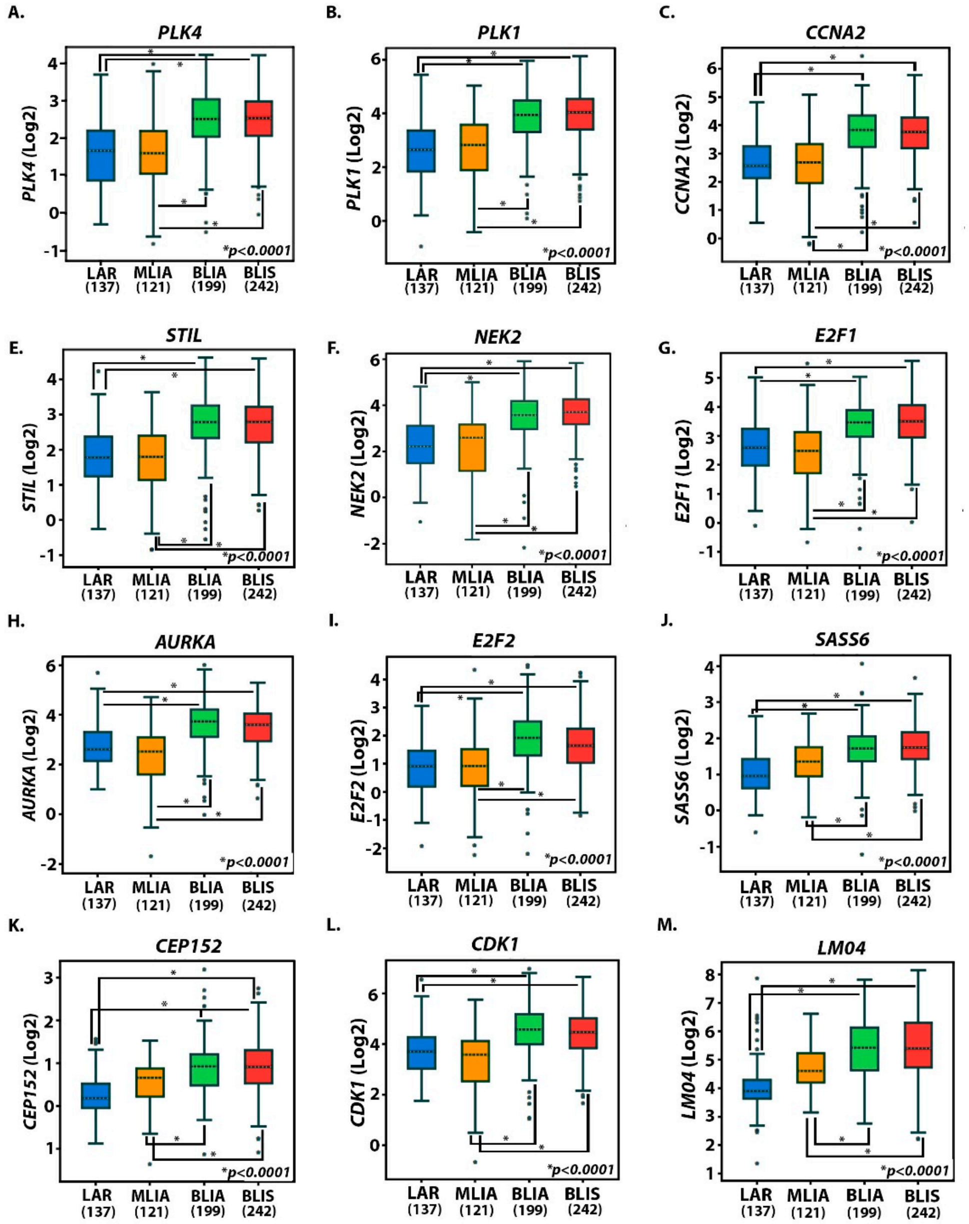

Figure S2. Differences in expression levels of centrosome-amplification genes among TNBC subtypes. RNA level differences of centrosome-amplification genes between the four different TNBC subtypes in bc-GenExMiner including LAR, luminal androgen receptor; MLIA, mesenchymal-like immune activated; BLIA, basal-like immune activated; BLIS, basal-like immune suppressed.

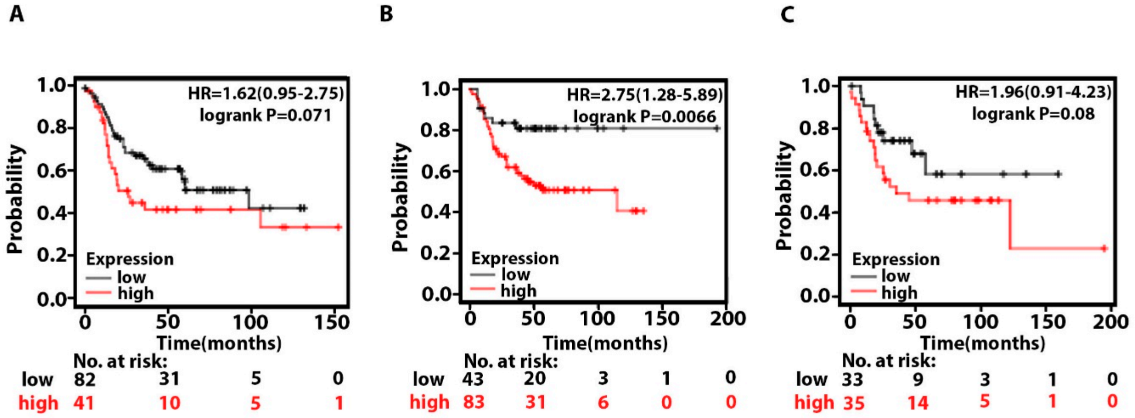

Figure S3. Association between expression of centrosome amplification-driving genes and recurrence-free survival in women with TNBC. Kaplan-Meier associations of the mean expression of centrosome amplification-driving genes with recurrence free-survival among LAR (A), BL1 (B), and BL2 (C) TNBC patients in KM Plotter breast cancer database. Optimal cut-offs were referred to for all Kaplan-Meier analyses. CDK1 gene was excluded from analyses due to lack of existing probe.
